# Supplementary material for: Murine Langerin+ dermal dendritic cells prime CD8+ T cells while Langerhans cells induce cross-tolerance
Source: EMBO Mol Med. 2014 Aug 1;6(9):1191–204. doi: 10.15252/emmm.201303283 (PMC4197865; doi:10.15252/emmm.201303283)
Supplement: Supplementary file 1 — Supplementary Figures [file emmm0006-1191-SD1.pdf]

EMBO Molecular Medicine

Murine Langerin+ dermal dendritic cells prime CD8+ T cells while Langerhans cells induce cross-tolerance.

Vincent Flacher, Christoph H. Tripp, David G. Mairhofer, Ralph M. Steinman, Patrizia Stoitzner, Juliana Idoyaga, and Nikolaus Romani

Supplementary Information - Table of Contents

Figure S1: Langerin targeting combined with imiquimod adjuvant fails to induce cytotoxic responses.

Figure S2: Distribution of targeting mAb is not changed by the addition of adjuvants

Figure S3: Differential expression of immunoregulatory receptors and IL-10 by DCs

Figure S4: Selective Langerin expression in DC subsets following irradiation and bone marrow transfer.

Figure S5: Langerin/OVA plus poly(I:C) and anti-CD40 yields tolerance only in the absence of dermal DC targeting.

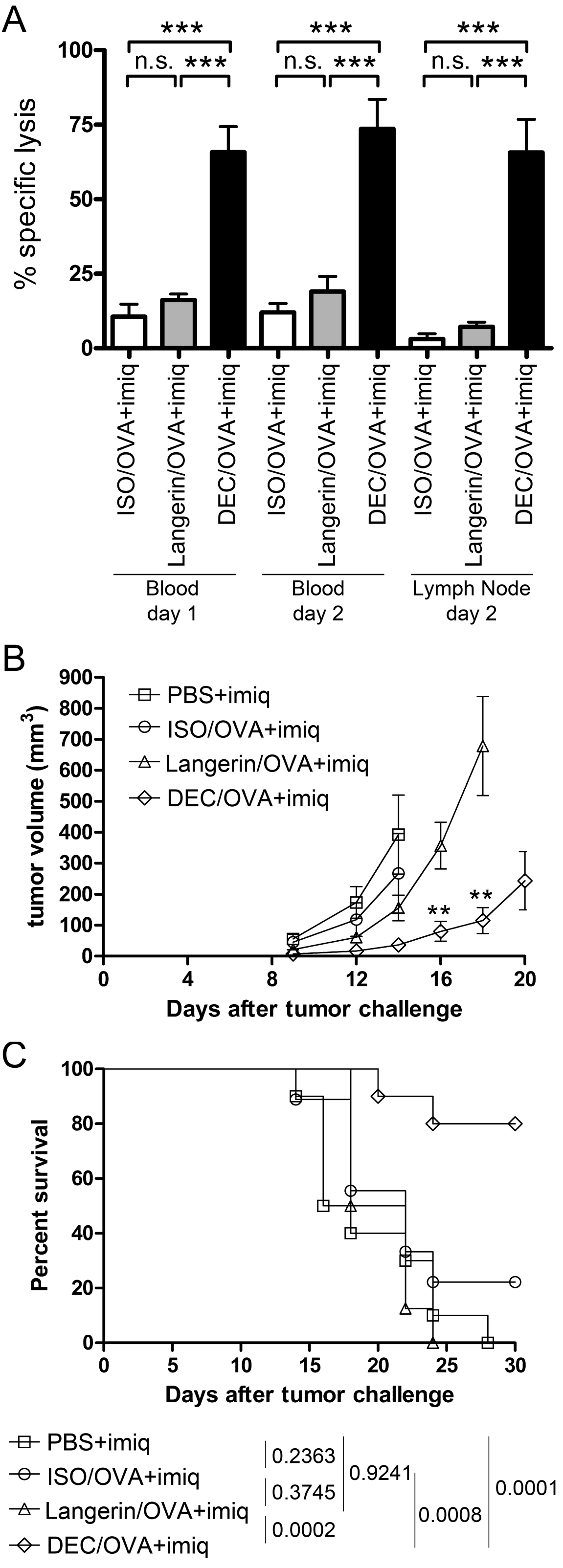

A
***
n.s.
% specific lysis
100
75
50
25
0
ISO/OVA+imiq
Langerin/OVA+imiq
DEC/OVA+imiq
Blood
day 1
Blood
day 2
Lymph Node
day 2
B
tumor volume (mm3)
900
800
700
600
500
400
300
200
100
0
PBS+imiq
ISO/OVA+imiq
Langerin/OVA+imiq
DEC/OVA+imiq
**
0
4
8
12
16
20
Days after tumor challenge
C
Percent survival
100
80
60
40
20
0
0
5
10
15
20
25
30
Days after tumor challenge
PBS+imiq
ISO/OVA+imiq
Langerin/OVA+imiq
DEC/OVA+imiq
0.2363
0.3745
0.0002
0.9241
0.0008
0.0001

Figure S1

Figure S1: Langerin targeting combined with imiquimod adjuvant fails to induce cytotoxic responses.

(A) In vivo killing of OVA-loaded target cells. C57BL/6 mice were immunized i.d. into both ears with 0.5µg ISO/OVA isotype control, Langerin/OVA or DEC/OVA in addition to topical imiquimod application (+imiq). Seven days later, CFSE-labeled OVA-loaded target cells and CTO-labeled unloaded control cells were transferred i.v. Specific lysis of OVA-loaded target cells was measured in the blood one day later, and in both blood and ear-draining lymph nodes after two days. Data of individually analysed mice are pooled from two independent experiments (ISO/OVA+imiq: 6 mice; Langerin/OVA+imiq: 4 mice; DEC/OVA+imiq: 6 mice) and compared using one-way ANOVA ($p<0.0001$) followed by Tukey's test (n.s. : non significant, $p>0.05$). **(B-C)** Tumor protection assay. C57BL/6 mice were immunized i.d. into both ears with PBS or 0.5µg ISO/OVA isotype control, Langerin/OVA or DEC/OVA in addition to topical imiquimod application (+imiq). One month after immunization, 10^5 B16 melanoma cells expressing OVA were implanted subcutaneously. Tumor growth **(B)** and survival of the recipient mice **(C)** were monitored three times a week. Data are pooled from two independent experiments (PBS+imiq: 9 mice; ISO/OVA+imiq: 10 mice; Langerin/OVA+imiq: 8 mice; DEC/OVA+imiq: 9 mice). Tumor growth was compared using Student's unpaired t-test (day 16, $p=0.0030$; day 18, $p=0.0026$). Survival curves were compared using a Mantel-Cox test.

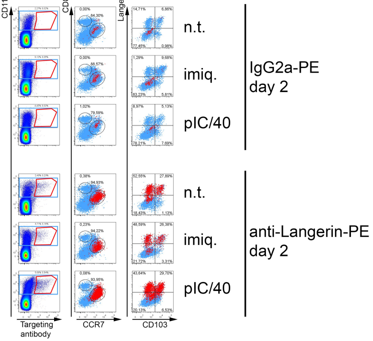
CD11c
CD8
Langerin
0,00%
84,30%
14,71%
6,86%
77,45%
0,98%
n.t.
0,00%
88,57%
1,29%
9,68%
83,23%
5,81%
imiq.
1,02%
79,59%
8,97%
5,13%
78,21%
7,69%
pIC/40
IgG2a-PE
day 2
0,38%
94,93%
52,55%
27,89%
18,43%
1,13%
n.t.
0,23%
94,22%
48,59%
26,38%
21,72%
3,31%
imiq.
0,08%
93,95%
43,64%
29,70%
20,13%
6,53%
pIC/40
anti-Langerin-PE
day 2
Targeting
antibody
CCR7
CD103

Figure S2

Figure S2: Distribution of targeting mAb is not changed by the addition of adjuvants

0.5µg of PE-coupled full-length anti-Langerin L31 antibody or rat IgG2a isotype control were injected intradermally into each ear of C57BL/6 mice, left untreated (n.t.) or with adjuvants imiquimod (imiq) or poly(I:C)+anti-CD40 (pIC/40). Two days later, mice were sacrificed and auricular lymph nodes collected, digested and analysed by flow cytometry. Left column: Gating strategy for total CD11c+ (Blue line) or targeted CD11c+ (Red line). Targeted cells appear as red dots in the following columns. Percentages show the proportion of targeted cells in a given quadrant or gate. Results are representative of two independent experiments.

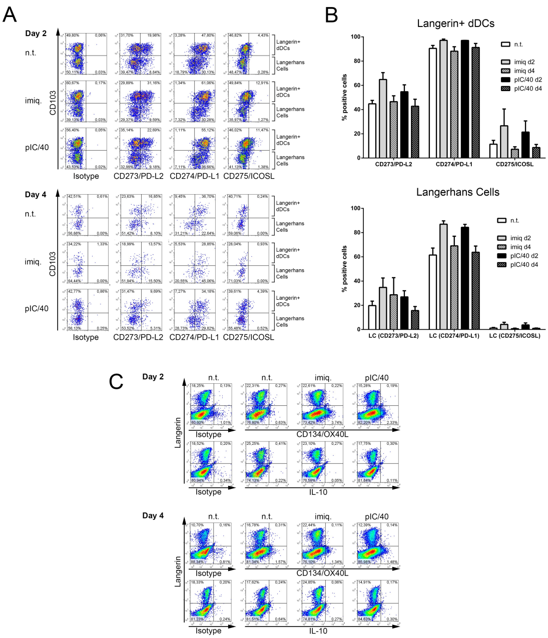
A
B
C
Day 2
Day 4
n.t.
imiq.
pIC/40
CD103
Isotype
CD273/PD-L2
CD274/PD-L1
CD275/ICOSL
Langerin+ dDCs
Langerhans Cells
% positive cells
imiq d2
imiq d4
pIC/40 d2
pIC/40 d4
LC (CD273/PD-L2)
LC (CD274/PD-L1)
LC (CD275/ICOSL)
Langerin
CD134/OX40L
IL-10

Figure S3

Figure S3: Differential expression of immunoregulatory receptors and IL-10 by DCs

Both ears of C57BL/6 mice were treated with imiquimod (imiq), poly(I:C) and anti-CD40 (pIC/40), or left untreated (nt). Two or four days later, a cell suspension was obtained from four auricular lymph nodes of two similarly treated mice and analysed by flow cytometry. Measurements, each comprising cells from two mice, are pooled from two independent experiments **(A)** Representative surface stainings and **(B)** percentages of cells expressing CD273/PD-L1, CD274/PD-L2, CD275/ICOSL in CD11c+ Langerin+ CD103^{neg} LCs and CD11c+ Langerin+ CD103+ dDCs. None of the differences reached statistical significance (nt: n=7; imiq - day 2: n=3; imiq - day 4: n=4; pIC/40 - day 2: n=3; pIC/40 - day 4: n=4; One-way ANOVA: LCs, p=0.9809; Langerin+ dDCs, p=0.9802). **(C)** Surface staining for OX40-ligand and intracellular expression of IL-10 in CD11c+ Langerin-/+ DCs after a 3h incubation with Brefeldin A (nt: n=4; imiq - day 2: n=2; imiq - day 4: n=2; pIC/40 - day 2: n=2; pIC/40 - day 4: n=2).

.

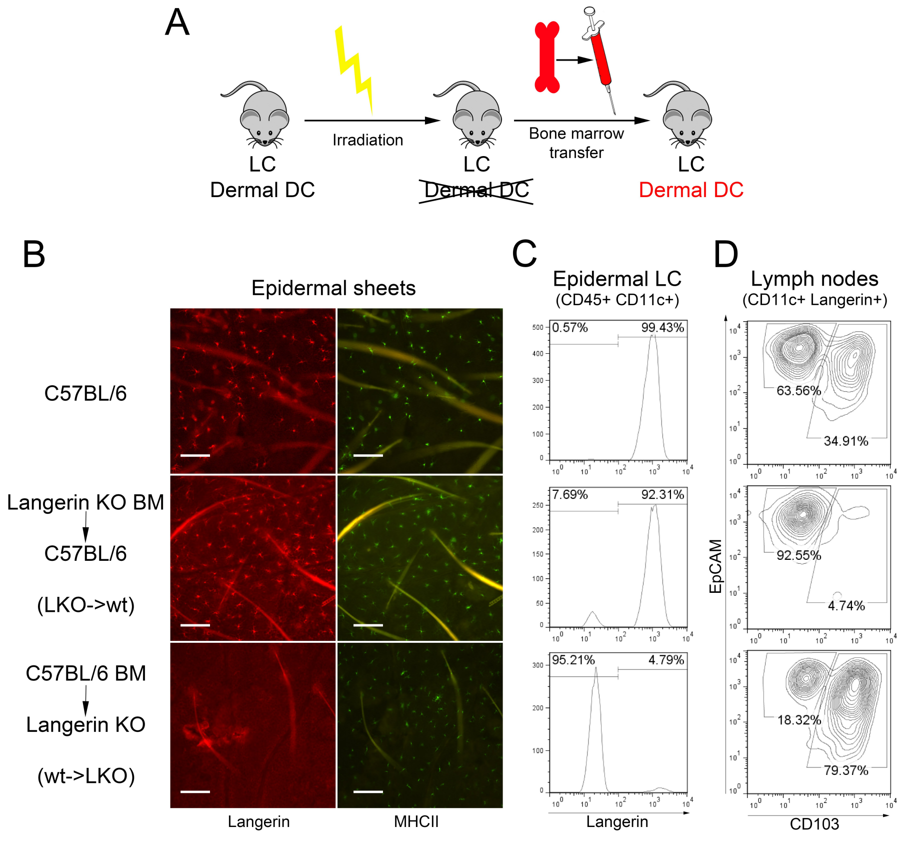
A
Irradiation
Bone marrow
transfer
LC
Dermal DC
LC
Dermal DC
LC
Dermal DC
B
Epidermal sheets
C57BL/6
Langerin KO BM
C57BL/6
(LKO->wt)
C57BL/6 BM
Langerin KO
(wt->LKO)
Langerin
MHCII
C
Epidermal LC
(CD45+ CD11c+)
0.57%
99.43%
7.69%
92.31%
95.21%
4.79%
Langerin
D
Lymph nodes
(CD11c+ Langerin+)
63.56%
34.91%
92.55%
4.74%
18.32%
79.37%
EpCAM
CD103

Figure S4

Figure S4: Selective Langerin expression in DC subsets following irradiation and bone marrow transfer.

C57BL/6 or Langerin$^{-/-}$ mice were lethally irradiated with a total dose of 10 Gy. Immediately after irradiation, 5x10^6 bone marrow cells from Langerin$^{-/-}$ or C57BL/6 were transferred intravenously, respectively. 8-12 weeks later, ear skin and skin-draining lymph nodes were obtained from chimeras. **(A)** Principle of irradiation/reconstitution experiments and their consequences on skin dendritic cells. **(B)** Epidermal sheets were stained with anti-Langerin Ab, followed with chicken anti-rat Ig/Alexa 594, and counterstained with anti-MHCII/FITC. Scale bars represent 100µm. **(C)** Langerin expression by CD45+ CD11c+ cells from digested epidermis. **(D)** CD11c+ Langerin+ lymph node DCs were stained for CD103 and EpCAM. Data are representative of eight reconstitution experiments.

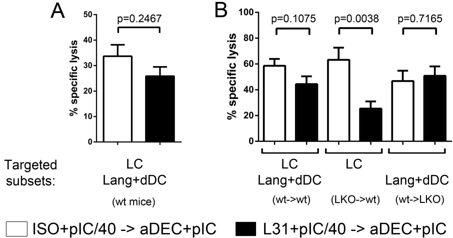

A
p=0.2467
% specific lysis
50
40
30
20
10
0
B
p=0.1075
p=0.0038
p=0.7165
% specific lysis
100
80
60
40
20
0
Targeted subsets:
LC
Lang+dDC
(wt mice)
LC
Lang+dDC
(wt->wt)
LC
(LKO->wt)
Lang+dDC
(wt->LKO)
ISO+pIC/40 -> aDEC+pIC
L31+pIC/40 -> aDEC+pIC

Figure S5

Figure S5: Langerin/OVA plus poly(I:C) and anti-CD40 yields tolerance only in the absence of dermal DC targeting.

Both ears were immunized with Langerin/OVA or isotype control ISO/OVA and treated with poly(I:C) and anti-CD40 (pIC/40). One week later, mice were injected i.p. with anti-DEC-205/OVA and poly(I:C) (DEC/OVA+pIC). Two weeks after the primary immunisation, CFSE-labeled OVA-loaded target cells and CTO-labeled unloaded control cells were transferred i.v. Specific lysis of OVA-loaded target cells was measured in the blood one day later. Data are pooled from two independent experiments and compared using Student's unpaired t-tests. **(A)** C57BL/6 mice (ISO+pIC/40→aDEC+pIC: 3 mice; L31+pIC/40→aDEC+pIC: 3 mice); **(B)** Chimeric mice: data from individually analysed mice are pooled from three independent experiments and compared using Student's unpaired t-test (wt→wt: [ISO+pIC/40→aDEC+pIC]: 7 mice, [L31+pIC/40→aDEC+pIC]: 8 mice; LKO→wt: [ISO+pIC/40→aDEC+pIC]: 8 mice, [L31+pIC/40→aDEC+pIC]: 8 mice; wt→LKO [ISO+pIC/40→aDEC+pIC]: 5 mice; [L31+pIC/40→aDEC+pIC]: 5 mice).
